# Case of Poison from a Vegetable Fungus; Together with Remarks on Professor Rossi's Two Cases of Supposed Rabies Canina

**Published:** 1804-11-01

**Authors:** Samuel Argent Bardsley

**Affiliations:** Physician to the Manchester Infirmary


THE
Medical and Phyfical Journal.

VOL. XII.] NOVEMBER 1, 1804. [NO. LXIX.

*Printed for R. PHILLIPS, by W. Thorne, Red Lion Court, Fleet Street, London.*

### CASE OF POISON FROM A VEGETABLE FUNGUS; *together with Remarks on Professor* ROSSI'S *two Cases of* SUPPOSED *Rabies Canina; by* SAMUEL ARGENT BARDSLEY, M.D. *Physician to the Manchester Infirmary.*

To the EDITORS of the MEDICAL and PHYSICAL JOURNAL.

Gentlemen,

I Trust you will consider the following case worthy of insertion in your widely-extended Journal, as it serves to point out one of the many unsuspected and frequently undiscovered causes of some alarming and anomalous disorders, which have perplexed practitioners in their treatment of children. I have no doubt, but great numbers of this class of patients fall a sacrifice to their ignorantly partaking of poisonous vegetables, both in a wild and cultivated state. Young children are prone, from hunger, curiosity, or diversion, to eat all kinds of vegetables which fall in their way; and ought, therefore, to be strictly watched and cautioned against so dangerous a practice. Whenever a healthy child becomes suddenly afflicted with the peculiar symptoms about to be described, it may be readily inferred, that some vegetable poison has been conveyed to the stomach. This supposition will lead to the speedy adoption of those remedies which are best calculated to save the life of the patient.

On the 29th of last month, I was called upon, at six in the evening, to visit Master S. aged five years, the son of a gentleman living at Ardwick, near this town. His parents informed me, that he went out to play in perfect health, after eating a moderate dinner, with a companion of nearly his own age, in the fields adjoining the town; and and in about two hours was led home in a state of alarming illness. He seemed to stagger like a person intoxicated, and with odd gesticulations, laboured to express his sufferings, but was unable to articulate a single syllable. When I saw the patient, which was probably about two hours after his first seizure, he appeared partially delirious, and uttered faint and indistinct screams. His pulse was slow, small, and somewhat irregular. The pupils of both eyes were much dilated, and the vision evidently imperfect. He seemed very averse to lying down, and his restlessness and impatience led him to make frequent attempts to walk about the room, but without any fixed object or design. His gait and gestures were those of a person inebriated. He was unable to answer any questions, or to express his feelings by words. Slight convulsive motions might be perceived in the legs and arms, which gradually extended to the muscles of the trunk, and produced irregular distortions of the whole body. The upper extremities began to swell, and assumed a livid colour; and the abdomen felt hard, and rather tumid. From the peculiarity and suddenness of the attack, I was led to conjecture, that the patient had swallowed some poisonous vegetable substance. But the fact could not be ascertained at the time, as his companion was far from being well, and too much alarmed and confused to give any satisfactory information. No time, however, was to be lost. The spine and extremities were rubbed with a volatile linniment (which happened to be at hand) until a warm bath, and stimulating glyster, could be prepared. He was almost *immediately* placed in a bath, of the temperature of 100 degrees, (the warm water being in readiness at a neighbouring dye-house) where he was suffered to remain for the space of ten minutes. On getting into bed, the glyster was administered. Pills, with calomel and extract of jalap, were soon after got down. A profuse sweating come on, which was supported by supplying the patient with lemon-whey, and other warm diluents. In about twenty minutes from the exhibition of the glyster, a copious stool was procured. The patient, who had become more tranquil from his first entrance into the bath, seemed *now* to be greatly relieved. Soon after, a vomiting of an offensive and greenish-coloured fluid supervened; and this operation was followed by a plentiful discharge from the bowels (but nothing could be discovered in either of the evacuations, which might serve to strengthen the supposition of the patient having swallowed any deleterious substance). An evident abatement abatement of the most untoward symptoms *immediately* succeeded. The dilatation of the pupils had almost disappeared. His pulse became firmer, and was increased from 70 to 90 beats in a minute. The patient was likewise able to articulate with tolerable distinctness; but he seemed like a person just roused from a long and deep sleep, unconscious of any thing that had happened to him. I directed the purging pills to be repeated during the night, until a complete evacuation of the bowels had taken place. The next morning, I found my patient, with the exception of some degree of languor and debility, entirely recovered from this severe attack. Upon strictly questioning him and his companion, it appeared, they had mistaken one of the fungi, a *species* of the *agaric*, for the mushroom; and that my patient had eaten a considerable quantity of this poisonous vegetable, while in the fields. His companion had eaten a smaller portion, and therefore escaped with, comparatively, little injury. If timely assistance had not been administered, this child would, most probably, have fallen a sacrifice to a fatal, and not uncommon mistake, with children of his age. It may not be improper to remark, that if I had been called to this patient at the commencement of this malady, I should have thought it highly expedient to have prescribed *an emetic*; but considering that sufficient time had elapsed, provided any vegetable poison had been introduced into the stomach, to admit of its passage into the bowels, I directed my first efforts to procure a plentiful evacuation by stool.

Permit me, Gentlemen, to subjoin the following observations and comments, which have been suggested by the perusal, (in a late number of your Journal) of Professor Rossi's reports on the efficacy of Galvanism, in two cases of *supposed* rabies canina. The fatal malignity of this disorder, which has hitherto baffled the skill and sagacity of our most eminent practitioners, will, I trust, sufficiently apologize for an attempt to investigate how far these reports may be entitled to confidence and approbation. Rational scepticism, on medical reports, ought to be entertained by every medical person, who feels a due sense of the utility and importance of the healing art; but especially with regard to those statements, in which it has been asserted, that diseases of the most *refractory* and *incurable* kind have yielded to remedies, either before unknown, or revived from the authority of obselete and neglected empirical records. The instances, in which the power of several remedies, in the treatment of formidable diseases, has been

 greatly greatly exaggerated, are too recent and notorious to require any mention to be made of them here. Hence the necessity of receiving with due caution reports of a similar kind, for a too easy credulity is liable to be converted into obstinate scepticism; and thus the medical art sustains a serious injury. How many valuable remedies have sunk into unmerited neglect, from the injudicious commendations bestowed upon them by over-sanguine and sometimes interested reporters! Unfortunately, an attachment to the marvellous, which possessed the older systematic writers of the continent, when treating on the subject of hydrophobia, seems to have been transmitted *unimpaired* to their medical successors. Even the accurate Morgagni has mistaken totally distinct diseases for the genuine hydrophobia, as his quotatious from Marcellus Donatus abundantly prove.* Indeed, if we were to give implicit credit to the reports of modern French and Italian authors, on the cure of hydrophobia, there is no complaint more effectually and readily to be subdued.

But when these statements are cautiously examined, there will appear just grounds for suspicion, that some other diseases have been confounded with canine madness; for an aversion to, and difficulty in, swallowing liquids, are often the concomitants of hysteria, and occasionally are to be met with in tetanus, and other spasmodic diseases; and likewise not unfrequently arise, from the influence of imagination † operated upon by terror, in those persons who have had reason to believe, that they were bitten by a rabid animal. I am afraid these remarks but too justly apply to the supposed cases of hydrophobia, under consideration. From my own observation of three fatal cases of *rabid* hydrophobia, (two of which fell under my immediate care) and from an attentive and diligent inquiry into the nature and history of this disease, I am induced to believe, that Professor Rossi's cases do not belong to those of *genuine* hydrophobia, or canine madness. I shall now proceed to point out the *defective testimony* in the statement of these reports, as well as the *want of accuracy*, in the conclusions drawn from them. It would indeed

* See Miscellaneous Observations on Canine and Spontaneous Hydrophobia (by S. A. Bardsley, M. D.) in the fourth Volume of the Manchester Memoirs.

† See Hamilton on Hydrophobia, and the 4th volume of the Manchester Memoirs.

indeed have been more satisfactory to have perused the original communication; but I must take it for granted, that every material circumstance in the history of these cases, has been transcribed from the author's account. It is of importance, in the first place, to observe, that no proofs are adduced in these reports of the actual madness of the dogs, at the time the patients sustained the injury. Persons, who are bitten by dogs, too readily admit the presumption of the animal's madness, and neglect the proper means to ascertain so important a circumstance. But even if the fact be admitted, it does not necessarily follow, that the infection must take place. For it is notorious, that not one dog in forty, supposed to be mad, is really so affected; and moreover it is a fact, founded on the observation of a great number of instances, that upon the average, not more than one person * out of twenty-five that have been exposed to the bite of dogs unquestionably rabid, has become infected with the disease. It is likewise of consequence to remark, how strongly the first patient laboured under the *impression* of *terror* from the nature of the accident; and also that he had cauterized himself with boiling oil, and the actual cautery, in order to excite a long suppuration. What degree of inflammation and irritation were excited in the parts thus treated, is not stated; but it is not unreasonable to imagine, it must have been very considerable, and equal to the production of the very acute pain in the patient's neck, from which he was relieved by some internal composing medicines. But another train of symptoms followed, which certainly denote a general derangement of the system: such as, a severe pain in the head, and dizziness, (which were relieved by an emetic.) These were succeeded by violent pains in most of the joints, particularly in those of the neck and back; and, finally, the patient was seized with terror at the sight of water, attended with convulsions of the lower jaw; and it is further stated, that he felt, as the disease advanced, a propensity *to bite*. With the exception of the hydrophobic symptoms, there does not appear in this description of the disease, any characteristic traits of canine madness. Great stress, indeed, seems to be laid on the *disposition to bite*, as a symptom peculiarly characteristic of the disease: Yet so far is the "cupiditas mordendi" (which enters into

* See Dr. Hunter's Paper in the Transactions for improving Medical Knowledge, vol. I. p. 295.

the the definition of *hydrophobia rabiosa* of Cullen, and other systematic writers) from being a pathognomonic symptom of canine madness, that it very rarely occurs, and then only at the close of the disease, when the patient, exhausted by watchfulness, impatience, and anxiety, falls into a temporary delirium. That this symptom depends merely on an *association of ideas*, strongly impressed upon the imagination, during the temporary delirium, is clearly proved from the fact of its never occurring in those patients who are ignorant of the nature of their malady; and consequently whose minds are not liable to be impressed with notions of the disease having originated from the bite of a rabid animal. The last victim of this dreadful malady, who fell under my care about ten months ago, died *unconscious of the nature* of his complaint, and never discovered the slightest inclination to *bite*, or imitate any of the actions* of the animal which had caused his sufferings. I have no hesitation, therefore, in ascribing the symptoms, exhibited by Professor Rossi's first patient, to the power of imagination, operating upon a nervous and irritable temperament; and which was greatly assisted by the severe treatment pursued, with a view to excite a long suppuration. That hydrophobia, or a dread of water, has been excited by other causes than the poison of a rabid animal, is too well known to admit of dispute. Medical authors abound with histories of supposed canine madness, which may be referred, with great propriety, to mania, solely excited by anxiety and terror. Indeed, it is always difficult to divest the mind of fear, when a bite has been received from an animal supposed io be mad. The case † of the clergyman, near Manchester, strikingly exemplifies the power of imagination, in producing the symptom of hydrophobia. This gentleman experienced the greatest dread of water, from merely visiting one of his parishioners affected with canine madness: nor was he relieved from this notional hydrophobia, but with great exertion and difficulty. In addition to the conflicts of the mind, it appears that P. Rossi's patient suffered from great local irritation of the body.

Two examples are upon record of persons dying under hydrophobic symptoms, from having wounded their own fingers,

* I may remark here, that the barking of hydrophobic patients, which authors have mentioned, and copied from each other, is merely occasioned by the effort of hawking or coughing up the viscid saliva, which incessantly troubles the patient in this disease.

† See Hamilton on Hydrophobia.

fingers, by biting them in a paroxysm of anger.* May not then the combined influence of bodily and mental irritation be considered as the chief, if not the sole causes of this patient's hydrophobic symptoms? — The truth of the supposition will be further strengthened, when we consider the total absence, in this case, of some of the most characteristic marks of canine madness; such as, the desire of solitude; a copious flow of viscid and ropy saliva; stricture at the throat, with occasional apprehensions of immediate suffocation; restlessness of the body, and a marked aversion to a recumbent posture. Now, all these symptoms, in a greater or lesser degree, invariably accompany every instance of *rabid* hydrophobia.

* See Mem. de la Soc. Roy de Medicine, Paris, Ann. 1783, Comment & de Boerhav. Aphorism.

